# Antimicrobial Activity of Naturally Occurring Phenols and Derivatives Against Biofilm and Planktonic Bacteria

**DOI:** 10.3389/fchem.2019.00653

**Published:** 2019-10-01

**Authors:** Danica J. Walsh, Tom Livinghouse, Darla M. Goeres, Madelyn Mettler, Philip S. Stewart

**Affiliations:** ^1^Chemistry and Biochemistry, Montana State University, Bozeman, MT, United States; ^2^Center for Biofilm Engineering, Montana State University, Bozeman, MT, United States

**Keywords:** biofilm, antimicrobial, anti-biofilm, biofilm inhibition, essential oil

## Abstract

Biofilm-forming bacteria present formidable challenges across diverse settings, and there is a need for new antimicrobial agents that are both environmentally acceptable and relatively potent against microorganisms in the biofilm state. The antimicrobial activity of three naturally occurring, low molecular weight, phenols, and their derivatives were evaluated against planktonic and biofilm *Staphylococcus epidermidis* and *Pseudomonas aeruginosa*. The structure activity relationships of eugenol, thymol, carvacrol, and their corresponding 2- and 4-allyl, 2-methallyl, and 2- and 4-*n*-propyl derivatives were evaluated. Allyl derivatives showed a consistent increased potency with both killing and inhibiting planktonic cells but they exhibited a decrease in potency against biofilms. This result underscores the importance of using biofilm assays to develop structure-activity relationships when the end target is biofilm.

## Introduction

*Pseudomonas aeruginosa* is a Gram-negative, rod shaped bacterium with a pronounced tendency to form biofilms. It is also an opportunistic pathogen that exhibits multidrug resistance (Hubble et al., 2018). Its ubiquity in hospital-acquired infection has provided impetus for advancements in treating infections and diminishing the number of associated illnesses. *Staphylococcus epidermidis* is a Gram-positive bacterium typically found on human skin and mucosa. This pathogen is known for causing infections in prosthetic joints and valves as well as in postoperative wounds and the urinary tract, due to catheter use. *S. epidermidis* is also among the five most common organisms found to cause hospital acquired infections (Sakimura et al., 2015). Unlike *P. aeruginosa, S. epidermidis* is typically a harmless commensal bacteria, although its ability to form biofilms increases its persistence on medical devices. With recent advances in understanding biofilm development, including molecular mechanisms and cell surface proteins of *S*. *epidermidis*, this opportunistic pathogen is gaining increased interest within the medical field (Büttner et al., 2015).

The majority of microorganisms in nature, including those responsible for hospital-acquired infections, live in association with surfaces as biofilms (Persat et al., 2015). Due to the secretion of proteins, extracellular DNA and polysaccharides, biofilm communities are encased in a robust matrix which reduces their susceptibility to antimicrobial agents as well as the immune system (Costerton et al., 1999; Donlan, 2001; Nadell et al., 2015; Otto, 2018). This poses a health concern due to the potential for these organisms to cause serious infections in patients with indwelling medical devices and those who are undergoing surgical procedures, stressing the need for novel methods in treating biofilm mediated infections (Richards and Melander, 2009; Xu et al., 2017). According to the Agency for Health care Research and Quality, hospital-acquired infections are in the top 10 leading causes of death in the United States, and are consequently responsible for nearly 100 thousand deaths per year (Collins, 2008). Several methods to prevent and inhibit biofilm formation have been proposed or implemented to varying degrees of success including chemical and physical modification of surfaces and application of antimicrobial compounds (Chmielewski and Frank, 2003; Cortés et al., 2011; Artini et al., 2017).

**Figure 1:**
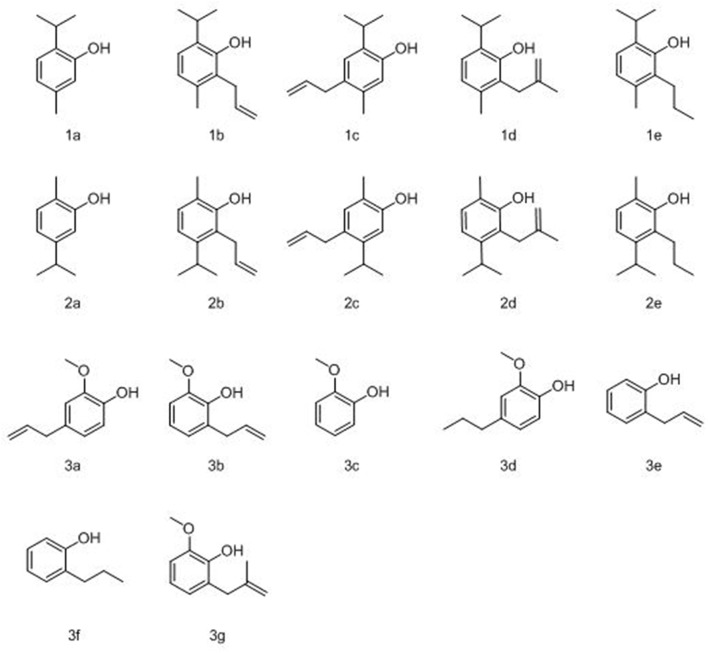
Structures of parent compounds, thymol (**1a**), carvacrol (**2a**), and eugenol (**3a**) as well as allyl (**1b/c**, **2b/c**, and **3b/e**), 2-methallyl (**1d, 2d**, and **3g**), and propyl (**1e**, **2e**, and **3d/f**) derivatives.

Phenols constitute an extensive class of compounds that have been shown to present antimicrobial properties against a wide range of bacteria (Lucchini et al., 1990; Cronin and Schultz, 1996; Puupponen-Pimia et al., 2001; Maddox et al., 2010; Alves et al., 2013; Shahzad et al., 2015; Villalobos Mdel et al., 2016; Pinheiro et al., 2018). Maddox et al. (2010) demonstrated that low-molecular weight phenolic compounds inhibit the growth of *X. fastidiosa*, a Gram-negative bacterium and plant pathogen, *in vitro*. Alves et al. (2013) studied phenolic compounds and their activity against *S. epidermidis, E. coli, Past. Multocida, N. gonorrhoeae*, MRSA, and several other Gram-negative and Gram-positive bacteria. Several essential oils have also been shown to present antimicrobial properties against taxonomically diverse bacteria both in planktonic and biofilm assays (Filoche et al., 2005; Ceylan and Ugur, 2015; Snoussi et al., 2015; Yang et al., 2015). This includes a variety of phenolic essential oils that have been studied as therapeutic and antimicrobial agents, such as thymol (**1a**), carvacrol (**2a**), and eugenol (**3a**) (Figure 1) which are plant metabolites (Juven et al., 1994; Shetty et al., 1996; Rasooli and Mirmostafa, 2003; Friedman, 2014; Marchese et al., 2017; Memar et al., 2017; Pinheiro et al., 2018). In a 2018 study, Pinheiro et al. (2018) studied thymol (**1a**) carvacrol (**2a**), eugenol (**3a**), “ortho-eugenol” (**3b**) and guaiacol (**3c**) as well as several chlorinated and allyl phenyl ether derivatives; these compounds were shown to be active toward several bacteria including *S. aureus* and *P. aeruginosa*. Eugenol has also been successfully evaluated for its antibacterial, antifungal, antiviral, anti-parasitic, and anti-cancer activity (Knobloch et al., 1989; Raja, 2015). In another study by Friedman (2014) several bioactivities of carvacrol (**2a**), including cell membrane disruptive properties, are extensively evaluated. This article also concludes that carvacrol has great potential to be used as a therapeutic for human infection and disease.

A number of structurally diverse essential oils, including thymol (**1b**), carvacrol (**2a**), and eugenol (**3a**) have been evaluated for their antimicrobial and anti-biofilm properties. Essential oils have been shown to act as biofilm inhibitors against Staphylococci (Al-Shuneigat et al., 2005; Noumi et al., 2018; Patsilinakos et al., 2019) as well as Pseudomonas (Carezzano et al., 2017; Farisa Banu et al., 2017; Artini et al., 2018). Thymol (**1a**) and carvacrol (**2a**) have demonstrated anti-biofilm properties, both alone and as a mixture, against diverse bacteria including *Cryptococcus* (Kumari et al., 2017)*, Salmonella* (Cabarkapa et al., 2019)*, Staphylococci* (Neyret et al., 2014)*, Enterococcus* (Pazarci et al., 2019), and *Escherichia* (Perez-Conesa et al., 2006). Eugenol (**3a**) has also been shown to exhibit anti-biofilm properties against a variety of Gram-negative and Gram-positive bacteria including *Porphyromonas* (Zhang et al., 2017)*, Salmonella* (Miladi et al., 2017)*, Escherichia* (Perez-Conesa et al., 2006), and *Listeria* (Liu et al., 2015).

The mechanism of action of several structurally varied naturally occurring phenols has been studied against a variety of microorganisms. The antimicrobial activity of essential oils has generally been attributed to a cascade of reactions involving the bacterial cell, as opposed to a single mode of action, which lead to degradation of the cytoplasmic membrane, damage of membrane proteins, reduced ATP synthesis, and increased membrane permeability (Knobloch et al., 1989; Lucchini et al., 1990; Lambert et al., 2001; Nazzaro et al., 2013). It has also been well-documented that the hydrophobicity of essential oils contributes to their antimicrobial activity by enabling them to disrupt the lipid bilayer in bacterial cells (Sikkema et al., 1994; Carson et al., 2002).

Carvacrol has been shown to destabilize the cytoplasmic membrane, increasing membrane fluidity causing leakage of ions, a decrease in the pH gradient across the cytoplasmic membrane and inhibition of ATP synthesis in *Bacillus cereus* (Ultee et al., 2002). The importance of the hydroxyl group on the aromatic ring in carvacrol has also been demonstrated by comparing carvacrol with similar compounds such as carvacrol methyl ester, methanol, and cymene; which lack the hydroxyl group that carvacrol possesses (Dorman and Deans, 2000; Ultee et al., 2002). Ultee et al. observed that carvacrol is able to diffuse through the cytoplasmic membrane, becoming deprotonated and then binding to a monovalent cation such as potassium it is able to diffuse out of the cytoplasm where it again takes up a proton from the external environment, there for acting as a transmembrane carrier of monovalent cations (Ultee et al., 2002). Another study by Knobloch et al. (1989) discusses the antimicrobial activity of essential oils as causing damage to the biological membrane. Knobloch et al. also speculated that the acidity of the hydroxyl group on thymol and carvacrol may attribute to their antimicrobial activity as well.

The effect of eugenol on the cell membrane has also been examined using *C. albicans* (Latifah-Munirah et al., 2015), showing that eugenol, like carvacrol, also targets the cytoplasmic membrane. Another study by Xu et al. (2016), demonstrated that eugenol disrupts the cell wall of *S. aureus*, increasing permeability, causing leakage of cellular substituents and permanent damage to the cell membrane. Eugenol has also been shown to bind to proteins in *E. aerogenes* and inhibit the production of enzymes in *B. cereus*, causing degradation of the cell membrane (Thoroski et al., 1989; Wendakoon and Sakaguchi, 1995).

A variety of phenolic essential oils and other aromatic alcohols that are not evaluated in this study, have also been studied for their mode of action. Wu et al. (2016) reported the antimicrobial activity and mechanism of action of the natural occurring phenol, 3-p-trans-coumaroyl-2-hydroxyquinic acid. In this study it was shown that this phenol caused the loss of cytoplasmic membrane integrity, increased membrane fluidity and caused conformational changes in membrane proteins of *S. aureus*. Aromatic alcohols such as phenoxyethanol have also shown to increase permeability of the cytoplasmic membrane in *E. coli* (Gilbert et al., 1977; Fitzgerald et al., 1992).

In this communication, thymol (**1a**), carvacrol (**2a**), and eugenol (**3a**) as well as guaiacol are evaluated along with several 2- and 4- allyl, 2-methallyl and 2-*n*-propyl derivatives (Figure 1). Thymol (**1a**), carvacrol (**2a**), and eugenol (**3a**) have also been evaluated for their ability to inhibit adherence and biofilm formation as well as biofilm eradication (Nostro et al., 2007; El Abed et al., 2011; Adil et al., 2014; Burt et al., 2014; Moran et al., 2014; Ceylan and Ugur, 2015; Kifer et al., 2016; Kim et al., 2016; Gaio et al., 2017; Lee et al., 2017; Miladi et al., 2017; Oh et al., 2017; Raei et al., 2017; Mohamed et al., 2018; Vazquez-Sanchez et al., 2018). Oh et al. (2017) has shown that thymol (**1a**) and carvacrol (**2a**) have anti-biofilm effects on the formation of *E. coli* and *Salmonella*. Unlike previous studies, the parent phenolic compounds are being compared to their allyl, methallyl, and propyl derivatives, which have not been extensively evaluated against either planktonic cells or biofilms.

Thymol (**1a**) and carvacrol (**2a**) are both monoterpenes and are constitutional isomers found in thyme, oregano, bergamot, and other culinary herbs (Figure 1). Both are used as a flavoring agents as well as in tinctures for their antifungal, antibacterial, and antiprotozoal properties (Escobar et al., 2010; Marchese et al., 2016). Eugenol (**3a**) is an essential oil found in plants such as vanilla, clove, nutmeg, and cinnamon. It is a flavoring agent utilized as well as for its antibacterial and anti-inflammatory properties (Marchese et al., 2017; Tsai et al., 2017). Guaiacol (**3c**) was also evaluated and is a naturally occurring phenol found in guaiacum, a shrub in the Zygophyllaceae family, and in creosote wood. It is structurally similar to eugenol, although it lacks the 4-allyl appendage. Guaiacol's ability to inhibit planktonic cell growth as well as biofilm formation in a mixture has also been evaluated (Cooper, 2013; Pinheiro et al., 2018). In this study, these four compounds and several of their derivatives have been assessed for potency toward inhibiting planktonic cell growth as well as their ability to eradicate biofilms.

The 2- and 4-allyl (**1b**, **2b, 1c**, and **2c)**, *n*-propyl (**1e** and **2e**) and 2-methallyl (**1d** and **2d**) derivatives of thymol and carvacrol as well as the 4-*n*-propyl derivative of eugenol (**3d**) and 2-allyl and 2-methallyl derivative of guaiacol (**3b** and **3g**) were evaluated, all of which are previously synthesized derivatives of these essential oils (Bartz et al., 1935; Lupo et al., 2000; Tsang and Brimble, 2007; Horáček et al., 2013) (Figure 1). None of these derivatives have previously been evaluated for their antimicrobial activity against biofilms. The corresponding allyl ether derivatives of thymol (**1a**) and carvacrol (**1b**) have been studied, Pinheiro et al. (2018) although to our knowledge the 2- and 4- allyl derivatives as well as 2-methallyl and 2-propyl derivatives have yet to be evaluated against both planktonic cells and biofilms in the same study. This study not only evaluates the potency of the derivatives stated above against planktonic cells but against biofilms as well, illustrating the difference in potency and trends in potency between these two modes of microbial growth. The structures that are being evaluated here are allyl, methallyl, or propyl groups and whether these groups increase potency of the selected essential oils. The addition of an allyl group was selected in effort to increase lipophilicity, and thus to increase permeability toward the cell membrane. Lacey and Binder (2016) also demonstrated that ethylene binds to an ethylene binding protein in Synechocystis affecting pili, which are binding proteins. The 2-methallyl group was also selected to increase lipophilicity.

The simple analogs 2-allylphenol (**3e**) and 2-*n*-propylphenol (**3f**) were also evaluated for comparative purposes to the aforementioned 2-allyl derivatives of the selected essential oils. The purpose of this investigation was to develop structure activity relationships for naturally occurring phenol derivatives and to compare these relationships between planktonic and biofilm modes of bacterial growth.

## Materials and Methods

### Experimental General Information

Thymol (**1a**) (99% pure), carvacrol (**2a**) (95% pure), guaiacol (99% pure), 2-allyl phenol (95% pure), and eugenol (**3a**) (99% pure) were purchased from Tokyo Chemical Industry Co. (TCI). All other reagents for chemical synthesis were purchased from commercial sources and used as received without further purification. Solvents for filtrations, transfers, and chromatography were certified ACS grade. Thin layer chromatography was performed on Silicycle Glass Backed TLC plates, and visualization was accomplished with UV light (254 nm), and/or potassium permanganate. All ^1^H NMR spectra were recorded on a Bruker DRX300. All ^13^C NMR spectra were recorded on a Bruker DRX500, all NMR data was reported in ppm, employing the solvent resonance as the internal standard.

*Pseudomonas aeruginosa* (PA01 and PA015442) and *S. epidermidis* (35984) were obtained from American Type Culture Collection (ATCC). All bacteria were sub-cultured onto tryptic soy agar (TSA) plates and incubated at 37°C for 24 h. Single colonies were transferred from the plates and inoculated into 25 mL tryptic soy broth (TSB) in Erlenmeyer flasks. Culture were incubated 37°C for 24 h and 10 μL of culture was transferred into 25 mL of TSB and the absorbance was read at 600 nm using a spectrophotometer and standardized to 10^6^-10^7^ CFU/mL.

### Efficacy of Naturally Occurring Phenols and Derivatives on Inhibiting Planktonic Cells

The minimum inhibitory concentrations (MICs) of all compounds against *S. epidermidis* and *P. aeruginosa* were determined using a 96-well plate assay previously described by Xie et al. (2012). The data from at least three replicates were evaluated for each compound tested. Samples were diluted in dimethyl sulfoxide (DMSO) and DMSO controls were conducted as the negative control. Experiments were done in biological triplicate and technical duplicates were done. Tests for statistical significance were calculated with a two-tailed *t-*test assuming unequal variances.

### Efficacy of Naturally Occurring Phenols and Derivatives on Killing Planktonic Cells

Parent compounds (**1a**, **2a**, and **3a**) were used as reference standards for each synthesized derivative. Both strains were cultured as described above. Compounds were diluted in 9.9 mL Phosphate-buffered saline (PBS) and 0.1 mL DMSO. Each tube was inoculated and allowed to sit at room temperature for 5 h, with sampling every hour. For sampling, three 10-fold dilutions were made in PBS. Each dilution was drop platted using 50 μL. Plates were incubated for 24 h and colony forming units (CFU) were counted. The concentration which showed no CFUs after 5 h was established as the lowest concentration which allowed for no bacterial growth. Negative controls with 9.9 mL PBS and 0.1 mL DMSO were done as well. Experiments were done in biological triplicate and technical duplicates were done. Standard deviations were determined by calculating the standard deviation for data from triplicate experiments. The mean log reduction was also determined for each compound evaluated using the following equation:

$$\begin{array}{l} Log\;reduction=\log10\;\left(\frac{A}{B}\right) \end{array}$$

where, *A* is the average number of CFU before treatment and *B* is the average number of CFU after treatment.

### Efficacy of Naturally Occurring Phenols and Derivatives on Biofilms

#### Biofilm Eradication Concentration Assays

Parent compounds (**1a**, **2a**, and **3a**) were used as reference standards for each synthesized derivative. Both strains were cultured as described above and biofilms were grown in Costar polystyrene 96-well plates at 37°C. After 24 h of incubation, the planktonic-phase cells were gently removed and the wells washed three times with PBS. Wells were filled with 150 μL dilutions of the compound being evaluated. The 96-well plates were incubated for an additional 24 h at 37°C. The media was gently removed and each well filled with 150 μL PBS and the biofilm broken up through stirring with sterile, wooden rods. Three 10-fold dilutions of each sample were taken and drop plated on TSA plates and incubated for 24 h. The biofilm eradication concentration (BEC) was determined to be the lowest concentration at which no bacterial growth occurred. This procedure was modeled based on previously reported procedures according to Pitts et al. (2003). Negative controls were also conducted with 150 μL PBS in the absence of antimicrobial agent. Experiments were done in biological triplicate and technical duplicates were done. Tests for statistical significance were calculated with a two-tailed *t*-test assuming unequal variances.

#### Center for Disease Control (CDC) Biofilm Reactor Evaluation

The parent compound eugenol (**3a**) was used as a reference standards for the synthesized derivatives. A CDC biofilm reactor was also used to assess potency of compounds toward biofilms. American Society for Testing and Materials (ASTM) method E2562–17 which describes how to grow a biofilm in the CDC biofilm reactor under high shear and continuous flow, and ASTM method E2871–13, a biofilm efficacy test generally known as the single tube method were used for this procedure. Formation of 48 h biofilms in a CDC reactor was formed on glass coupons (4.02 cm^2^). A CDC reactor containing 340 mL of TSB (300 mg/L) was inoculated with 1 mL of a 3.21 × 10^8^ CFU/mL overnight culture of *P. aeruginosa* (PA015442), which was grown in TSB (300 mg/L) overnight. The biofilm was grown in batch condition at room temperature at 125 rpm for 24 h, and then for 24 h at room temperature under continuous flow with a feed rate of 11.25 mL/min at 125 rpm. The continuous feed TSB was 100 mg/L. Coupons were then sampled from the reactor in triplicate. The mean log reduction in viable biofilms cells exposed to each compound for 1 h was quantitatively measured according to ASTM method E2871–13. After coupons were removed from the CDC reactor they were rinsed and transferred to separate, 50 mL conical tubes and 4 mL of a 100 mM solution of the antimicrobial compound being tested in sterile PBS buffer was added. The tubes were incubated at room temperature under static conditions or 1 h. After 1 h 36 mL DE broth was added and the biofilm was disaggregated by a series of vortexing and sonicating for 30 s each in the order of v/s/v/s/v. Each sample was diluted 10-fold six times and the diluted samples were drop plated on (Reasoner's 2A agar) R2A agar plates, incubated overnight at 37°C and enumerated. Experiments were done in biological duplicate and technical duplicates were done. The mean log reduction was also determined for each compound evaluated using the following equation:

$$\begin{array}{l} Log\;reduction=\log10\;\left(\frac{A}{B}\right) \end{array}$$

where, *A* is the average number of CFU before treatment and *B* is the average number of CFU after treatment.

### Chemical Synthesis Procedures

#### Preparation of 2-(2-propen-1-yl)-6-(1-methylethyl)-3-methylphenol (1b). Representative Procedure

A 25 mL round-bottom flask equipped with a magnetic stirring bar was charged with thymol **1a** (751 mg, 5 mmol, 1 equiv) and anhydrous acetone (5 mL) was added. Finely pulverized potassium carbonate (1.4 g, 10 mmol, 2 equiv) was then added at room temperature with stirring. The reactant mixture was heated to reflux and allyl bromide (0.5 mL, 6 mmol, 1.2 equiv) was added. The reactant mixture was heated to reflux for 5 h. The resulting mixture was cooled and filtered through celite, washed with brine and concentrated in vacuo to remove solvent and the by-product of diallyl ether. The crude phenyl ether was dissolved in N,N-diethylaniline (2 mL) and heated to 200°C with stirring for 12 h. N,N-diethylaniline was subsequently removed by washing the mixture with 10% sulfuric acid and extracting with ethyl acetate. The residue was purified via column chromatography (25% EtOAc/Hexane for elution) to afford 742 mg (78%) of **1b** as a yellow oil. ^1^H NMR data taken in CDCl_3_ and analytical data included the following. ^1^H NMR (300 MHz, CDCl_3_) **1b**: δ 6.98 (d, *J* = 7.82 Hz, 1H), 6.77 (d, *J* = 7.82 Hz, 1H), 5.95 (m, 1H), 5.12 (m, 2H), 4.93 (s, 1H) 3.44 (d, *J* = 5.88 Hz, 2H), 3.16 (sept, *J* = 6.87 Hz, 1H) 2.26 (s, 3H), 1.24 (d, *J* = 6.87 Hz, 6H). C^13^ NMR (500 MHz, CDCl_3_) **1b**: δ 19.2 (CH_2_), 22.9 (CH_3_), 26.8 (CH_2_), 29.3 (CH), 115.6 (CH_2_), 124.3 (C), 128.8 (CH), 132.1 (C), 134.7 (CH), 137.3 (CH), 139.4 (C), 150.7 (C). H^1^ NMR (300 MHz, CDCl_3_) Spectral data and general procedures for compounds **1c**, **1d**, **2b**, **2c, 2d**, and **3b** can be found in the Supplementary Data.

#### Preparation of 2-(2-n-propyl)-6-(1-methylethyl)-3-methylphenol (1c). Representative Procedure

A 10 mL round-bottom flask was charged with 10% Pd/C (30 mg, 0.28 mmol, 0.1 equiv). The round-bottom flask was put under an atmosphere of hydrogen and 100% ethanol (2 mL) was added. 2-allylthymol (**1b**) (300 mg, 1.5 mmol, 1 equiv) was added at room temperature and the reaction was allowed to stir for 12 h. The resulting mixture was filtered through silica and concentrated in vacuo to afford 260 mg (87%) of **1e** as a light, yellow oil. ^1^H NMR data taken in CDCl_3_ and analytical data included the following. ^1^H NMR (300 MHz, CDCl_3_) **1e**: δ 6.95 (d, *J* = 7.88 Hz, 1H), 6.81 (d, *J* = 7.88 Hz, 1H), 2.98 (sept, *J* = 6.81 Hz, 1H), 2.56 (t, *J* = 7.89 Hz, 2H), 2.25 (s, 3H) 1.59 (q, *J* = 7.89, 7.32 Hz, 2H), 1.36 (d, *J* = 6.81 Hz, 6H) 0.96 (t, *J* = 7.32 Hz, 3H). C^13^ NMR (500 MHz, CDCl_3_) **1e**: δ 14.5 (CH_3_), 19.4 (CH_3_), 22.3 (CH_3_), 22.7 (CH_2_), 27.1 (CH_2_), 28.8 (CH_2_), 122.3 (CH), 123.1 (C), 126.5 (CH), 131.3 (CH), 134.7 (C), 150.8 (C). Spectral data and general procedures for compounds **2e**, **3d**, and **3f** can be found in the Supplementary Data.

## Results and Discussion

**Scheme 1:**
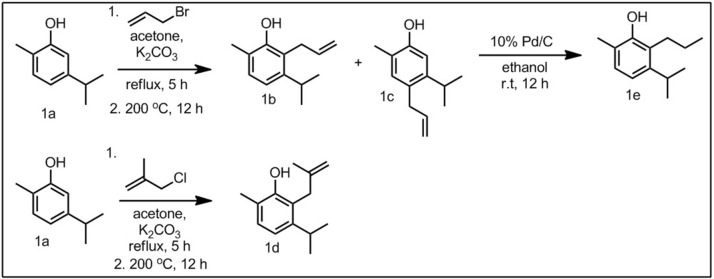
Representative synthesis, using carvacrol (**1a**) and derivatives (**1b−1d**).

In this study, four 2-allyl derivatives were synthesized, with the corresponding 4-allyl derivative as a secondary product (Scheme 1). This was accomplished through the synthesis of the corresponding allyl ether, followed by a thermal Claisen rearrangement. The allylated compounds **1b**–**3b**, **3a**, and **3e** were then hydrogenated to give the propyl derivatives (**1e**, **2e**, **3d**, and **3f**, Figure 1). Compounds **1a**, **2a**, and **3c** were also converted to the corresponding methallyl derivatives in an analogous manner.

In an initial study we assessed each naturally occurring phenol and its derivatives against planktonic cells. Studies have shown that thymol (**1a**) and carvacrol (**2a**) compromise the outer membrane of Gram-negative bacteria increasing the permeability of the cytoplasm (El Abed et al., 2011). The 2- and 4-allyl compounds for thymol (**1a**) and carvacrol (**2a**) all showed an increase in potency toward planktonic cells when compared to the parent compounds, as seen in Table 1. Interestingly, the 2-allyl derivatives (**1b** and **2b)** were more potent than the corresponding 4-allyl isomers (**1c** and **2c)** toward *P. aeruginosa*, whereas both isomers had identical MICs against *S. epidermidis* (Table 1). The transposed isomer “ortho-eugenol” (**3b)** was more potent toward both *S. epidermidis* and *P. aeruginosa* than the parent eugenol (**3a**). 4-*n*-propyl-2-methoxyphenol (**3d**) was more potent than eugenol (**3a**). Guaiacol (**3c**), which does not possess an allyl appendage was less potent toward *S. epidermidis* when compared to eugenol (**3a**) (Table 1). The methallyl derivate of carvacrol (**2d**) was more potent than the *n*-propyl derivative (**2e**) against *S. epidermidis*, though in the cases of methallyl thymol (**1d**) and methallyl eugenol (**3g**), the *n*-propyl derivatives (**1e** and **3f**) were more potent in comparison (Table 1).

**Table 1:** Minimum inhibitory concentrations in mM of parent compounds and derivatives against planktonic cells of *S. epidermidis* and *P. aeruginosa*.

	**MIC (mM)**	
**Compound**	***S. epidermidis* (35984)**	***P. aeruginosa* (PA01)**
Thymol (**1a**)	2.5	3.9
2-allylthymol (**1b**)	0.12	0.25
4-allylthymol (**1c**)	0.12	0.97
2-methallylthymol (**1d**)	15	31.2
2-*n*-propylthymol (**1e**)	7.8	15.62
Carvacrol (**2a**)	2.5	3.9
2-allylcarvacrol (**2b**)	0.12	0.25
4-allylcarvacrol (**2c**)	0.12	0.97
2-methallylcarvacrol (**2d**)	3.9	31.2
2-*n*-propylcarvacrol (**2e**)	7.8	15.62
Eugenol (**3a**)	15	31.2
ortho-eugenol (**3b**)	7.8	7.8
Guaiacol (**3c**)	31.2	31.2
2-methoxy-4-*n*-propylphenol (**3d**)	7.8	15.62
2-allylphenol (**3e**)	7.8	7.8
2-*n*-propylphenol (**3f**)	15.62	15.62
2-methallyl-4-methoxyphenol (**3g**)	62.5	125

Planktonic MICs of allyl derivatives were generally statistically significantly lower than the MIC of the parent compound. For example, the *p*-values for parent compounds and their allyl derivatives were also calculated. The *p*-value of 1a/b against *S. epidermidis* is 0.041 and against *P. aeruginosa* is 0.025. The *p*-value of 2a/b against *S. epidermidis* is 0.0003 and against *P. aeruginosa* is 0.0005. Likewise, the *p*-value of 3a/c against *S. epidermidis* is 0.016 although against *P. aeruginosa* was calculated to be 0.42 due to the similarities in potency.

**Figure 2:**
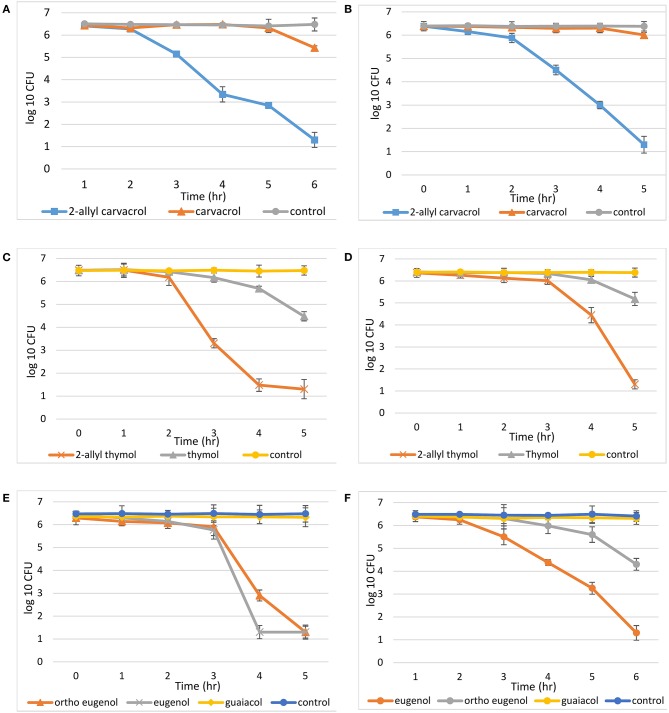
Time kill assays. Compounds were diluted in PBS and DMSO, (9.9:0.1), all controls were PBS and DMSO: **(A)** carvacrol, 2-allyl carvacrol, and a control with *S. epidermidis*, while the concentration of both carvacrol and 2-allyl carvacrol was 1.7 mM; **(B)** carvacrol, 2-allyl carvacrol, and a control with *P. aeruginosa*. While the concentration of both carvacrol and 2-allyl carvacrol was 15.6 mM; **(C)** thymol, 2-allyl thymol, and a control with *S. epidermidis*, at a concentration of 7.8 mM; **(D)** thymol, 2-allyl thymol, and a control with *P. aeruginosa*, at a concentration of 30 mM; **(E)** eugenol, “ortho eugenol,” guaiacol, and a control with *S. epidermidis*, at a concentration of 1.7 mM; **(F)** eugenol, “ortho eugenol,” guaiacol, and a control with *P. aeruginosa*, at a concentration of 15.6 mM.

Time dependent killing data against planktonic bacteria were measured for all 2-allyl and parent compounds (Figure 2). It was shown that over the time period of 5 h, 2-allyl carvacrol (**2b**) reduced bacterial growth by 79.80% against *S. epidermidis* and 79.63% against *P. aeruginosa*. The parent compound, carvacrol (**2a**), only reduced bacterial growth by 15.55% against *S. epidermidis* and 2.35% against *P. aeruginosa*. Similarly, 2-allyl thymol (**1b**) reduced the average bacterial growth by 79.00% for *S. epidermidis* and 77.93% for *P. aeruginosa*, while the average reduction of growth for thymol (**1a**) was 25.67% for *S. epidermidis* and 19.18% for *P. aeruginosa*. In the eugenol series, against *S. epidermidis*, eugenol (**3a**) and ortho eugenol (**3b**) had similar potency after 5 h with a decrease in bacterial growth of 79.76 and 79.34%, respectively. Although, at 4 h, eugenol (**3a**) was able to decrease growth by 79.76% while ortho eugenol (**3b**) decrease growth by 53.89%. Against *P. aeruginosa*, eugenol (**1a**) reduced growth by 79.60%, while ortho eugenol (**3b**) reduced growth by 32.88%. Against both bacteria, guaiacol (**3c**) reduced growth by <2%. On average, the controls for each study showed a 0.079% decrease in growth for *S. epidermidis* and a 0.45% decrease in *P. aeruginosa*.

The mean log reduction after 5 h was recorded for all evaluated compounds as well (Table 2). In this assay the 2-allyl derivatives of thymol and carvacrol (**1b** and **2b**) exhibited greater potency than the parent compound. Like 2-allylthymol and 2-allyl carvacrol, eugenol (**3a**) also exhibited a 5 log reduction after only 5 h, demonstrating that these allylate derivatives have bactericidal activity toward planktonic cells. Ortho-eugenol (**3b**) also exhibited a 5 log reduction against *S. epidermidis* after 5 h.

**Table 2:** Mean log reduction after 5 h against of exposure.

	***S. epidermidis***		***P. aeruginosa***	
**Compound**	**Mean log reduction**	**Concentration (mM)**	**Mean log reduction**	**Concentrations (mM)**
Thymol (**1a**)	1.9	7.8	1.2	30
2-allylthymol (**1b**)	5.1	7.8	5	30
Carvacrol (**2a**)	0.09	1.7	0.37	15.6
2-allylcarvacrol (**2b**)	5.1	1.7	5	15.6
Eugenol (**3a**)	5.2	1.7	5.1	15.6
Ortho-eugenol (**3b**)	2.6	1.7	2.1	15.6
Guaiacol (**3c**)	0.13	1.7	0.1	15.6

*Concentrations of thymol and allyl thymol against S. epidermis was 7.8 and 30 mM against P. aeruginosa, while carvacrol, 2-allylcarvacrol, eugenol, ortho-eugenol, and guaiacol was exposed at 1.7 mM against S. epidermidis and 15.6 mM against P. aeruginosa*.

The lower mean log reduction further conveys the inferiority of thymol (**1a**) and carvacrol (**2a**) to their 2-allyl derivatives in killing bacteria (Table 2). This observation is consistent with the MIC data presented in Table 1. In the dynamic killing assay, eugenol (**3a**) was more potent than both guaiacol (**3c**) and “ortho-eugenol” (**3b**) with a mean log reduction of 5.2 against *S. epidermidis* and 5.1 against *P. aeruginosa* (Table 2). This corresponds to a differing trend in activity when compared to the MICs in Table 1, where “ortho-eugenol” (**3b**) demonstrated a stronger growth inhibition than eugenol (**3a**).

Efficacious concentrations varied greatly between MICs and BECs. BECs were consistently higher than MICs, conforming to the expected lower susceptibility of bacteria in the biofilm mode of growth. In addition, biofilm assays exhibited significant differences in the structure-activity relationship in comparison to planktonic results. Thymol (**1a**) and carvacrol (**2a**) continued to show a higher potency than their 2-*n*-propyl derivatives **1e** and **2e** (Table 1), although they were more potent than their 2-allyl derivatives **1b** and **2b** against biofilms (Table 3). The 4-allyl derivatives **1c** and **2c** however, did have an identical or a slightly lower BEC, against *P. aeruginosa* and thus were still more potent than their 2-allyl counterparts.

**Table 3:** Biofilm eradication concentrations in mM of parent compounds and derivatives against *S. epidermidis* and *P. aeruginosa*.

	**BEC (mM)**	
**Compound**	***S. epidermidis***	***P. aeruginosa***
thymol (**1a**)	3.9	15.6
2-allylthymol (**1b**)	9.25	31.25
4-allylthymol (**1c**)	6.5	13
2-n-propylthymol (**1e**)	31.25	62.5
carvacrol (**2a**)	1.95	7.5
2-allylcarvacrol (**2b**)	9.25	31.25
4-allylcarvacrol (**2c**)	3.25	7.5
2-*n*-propylcarvacrol (**2e**)	31.25	62.5
eugenol (**3a**)	31.25	62.5
ortho-eugenol (**3b**)	31.25	31.25
guaiacol (**3c**)	7.8	15.6
2-methoxy-4-*n*-propylphenol (**3d**)	31.25	31.25

“Ortho-eugenol” (**3b**) continued to be more potent than eugenol (**3a**) against *P. aeruginosa* although the BECs for both compounds against *S. epidermidis* were identical. Guaiacol (**3c**) was the most potent against both bacteria in a biofilm when compared to other eugenol derivatives, which was dissimilar to the trend in potency against both killing and inhibiting planktonic cells (Table 1). The 4-*n*-propyl derivative of eugenol (**3d**) exhibited the same potency as “ortho-eugenol” (**3b**) against both bacteria (Table 3). It was interesting here that the MICs for the 4-*n*-propyl derivative **3d** were lower than eugenol (**3a**) against both bacteria tested, but the BEC against *S*. *epidermidis* was the same for both compounds (Table 3). This information illustrates that it is not reliable to predict structure activity relationships against biofilms based on planktonic cell data.

Unlike what was seen with MICs, the BECs of allyl derivatives were generally statistically significantly higher than the BECs of the corresponding parent compound. For example, the *p-*values for parent compounds and their allyl derivatives against biofilms were also calculated. The *p-*value of 1a/b were calculated to 0.022 be against *S. epidermidis* and 0.019 against *P. aeruginosa*. The *p-*value of 2a/b were calculated to be 0.009 against *S. epidermidis* and 0.023 against *P. aeruginosa*. The *p*-value of 3a/c is 0.003 against *S. epidermidis* and 0.015 against *P. aeruginosa*.

A CDC Biofilm reactor assay was also used to substantiate the comparative efficacy of thymol and its allyl and n-propyl derivatives against *P. aeruginosa* (PA015442) (Table 4). Here biofilms were grown in a high sheer environment as opposed to a static environment in 96-well plates as was done with BEC evaluations. This increases the biofilms adherence to the surface which it is grown. The CDC biofilm assay was chosen for this purpose. Methods were performed in accordance with ASTM; Designations: E 1054–08, E2562–17, and E2871–13. The *P. aeruginosa* strain (PA015442) used in this experiment was used because it was the strain named in the ASTM procedures. Results with the CDC biofilm reactor was consistent with the relative efficacies determined in the BEC assay (Tables 3, **4**). Thymol (**1a**) had the highest mean log reduction, correlating with the highest potency (Table 4). The 2-allylthymol (**1b**) was less potent than the parent and the *n*-propyl derivative (**1e**).

**Table 4:** Mean log reductions of thymol, 2-allylthymol, and 2-n-propylthymol against *P. aeruginosa* (PA015442).

**Compound**	**Mean log reduction**	**Concentration (mM)**
Thymol (**1a**)	4.48	100
2-allylthymol (**1b**)	0.13	100
2-*n*-propylthymol (**1e**)	0.21	100

MIC and BECs were also measured for strain PA015442 to compare with strain PA01 that was used with all other assays apart from the CDC biofilm reactor. As with strain PA01, 2-allylthymol (**1b**) exhibited the highest degree of potency against planktonic cells of PA015442 with an MIC of 0.08 mM, whereas thymol (**1a**) had an MIC of 0.68 (Table 5). It was interesting that thymol (**1a**), 2-allylthymol (**1b**) and 2-*n*-propylthymol (**1e**) were more effective against PA015442 (Table 5) than against PA01 in biofilm assays; whereas thymol (**1a**) had a BEC of 15 mM, 2-allylthymol (**1b**) had a BEC of 31.25 mM and 2-*n*-propylthymol (**1e**) a BEC of 62.5 mM against the PA01 strain (Table 3). In planktonic cell assays, the three compounds evaluated against PA015442 were also more potent toward PA01 (Tables 3, 5). Again, in accordance with previously observed BEC trends, thymol (**1a**) exhibited a lower BEC than both 2-allylthymol (**1b**) and 2-*n*-propylthymol (**1e**) as seen in Table 5.

**Table 5:** Biofilm eradication concentrations and minimum inhibitor concentrations of thymol, 2-allylthymol, and 2-n-propylthymol against *P. aeruginosa* PA015442, as well as mean log reduction at 100 mM.

	**Mean log reduction**	**MIC (mM)**	**BEC (mM)**
**Compound**	**PA015442**		
Thymol (**1a**)	4.48	0.68	1.37
2-allylthymol (**1b**)	0.13	0.08	6.5
2-*n*-propylthymol (**1e**)	0.21	3.125	25

The addition of an allyl group to the naturally occurring phenols thymol (**1a**) and carvacrol (**2a**) increased the compounds potency toward both inhibiting and killing planktonic cells; although decreased their ability to eradicate biofilms. Similarly, the elimination of an allyl group from the essential oil eugenol (**3a**) decreased potency toward planktonic cells and increased potency toward biofilms.

Naturally occurring phenols such as thymol (**1a**), carvacrol (**2a**), and eugenol (**3a**) have been shown to present antimicrobial properties against both planktonic cells and biofilms (Nostro et al., 2007; El Abed et al., 2011; Adil et al., 2014; Burt et al., 2014; Ceylan and Ugur, 2015; Kifer et al., 2016; Lee et al., 2017; Miladi et al., 2017; Raei et al., 2017; Mohamed et al., 2018). Here we demonstrated that 2-allyl (**1b** and **2b**) and 4-allyl derivatives (**1c** and **2c)** of thymol (**1a**) and carvacrol (**2a**) showed an increase in potency in comparison to the parent compounds against planktonic cells in both growth inhibition and killing assays. In biofilm assays the opposite trend was always observed: the non-allylated, parent compounds exhibited a higher potency than the allyl derivatives. Similarly, the non-allylated guaiacol (**3c**) was less potent against planktonic bacteria but more potent than eugenol (**3a**) or ortho-eugenol (**3b**) against biofilm bacteria. These observations underscore that structure-activity relationships determined for planktonic bacteria can be completely different than those for biofilms formed by the same species.

The fact that structure-activity relationships diverge between planktonic and biofilm assays may indicate that these compounds experience different limitations to their efficacy against planktonic and biofilm forms of the bacteria. It can be for example, that the penetrations of the agents into the biofilm is rate-limiting. Alternatively it could be that the permeability of the compounds into the cytoplasm of the cell becomes rate-limiting in the biofilm mode of growth. A third possibility is that the expression of molecular targets differs between planktonic and biofilm cells. If these mechanisms were better understood, it might be possible to rationally design superior anti-biofilm antimicrobial agents.

The 2-*n*-propyl derivatives (**1e** and **2e**) consistently were least potent compared to parent compounds and corresponding allyl derivatives. Here an allyl group will increase potency in comparison with a propyl group against both planktonic cells and biofilms. Both thymol (**1a**) and carvacrol (**2a**) have two alkyl groups, which are weakly electron donating. Eugenol (**3a**) in comparison has a methoxy group which is strongly electron donating as well as an allyl group; studies showed that eugenol (**3a**) was less potent than thymol (**1a**) and carvacrol (**2a**) in assays evaluating potency toward killing biofilms and inhibiting planktonic cell growth. Although was more efficacious toward killing planktonic cells, this is likely due to the presence of an allyl group.

Thymol (**1a**) and carvacrol (**2a**) are constitution isomers and had identical MICs against both *S. epidermidis* and *P. aeruginosa* (Table 1). The same was observed with their 2-allyl derivatives (**1b** and **2b**), as well as both 4-allyl derivatives (**1c** and **2c**) (Table 1). The 2-allyl derivative of thymol (**1b**) though, was less efficient in killing, as shown in Table 2, where the mean log reduction of thymol is 5.1 at 7.8 mM but the mean log reduction for carvacrol is 5.1 at 1.7 mM, although carvacrol (**2a**) was less potent than thymol (**1a**) (Table 2).

In biofilm eradication assays, carvacrol (**2a**) was more potent than thymol (**1a**) against both bacteria (Table 3). 4-allylcarvacrol (**2c**) was also more potent than 4-allylthymol (**1c**), although the 2-allyl derivatives exhibited the same BEC. Over all, there was very little difference in changing the allyl group from the 2 to the 4 position. The decreased potency of thymol against biofilms may result from the isopropyl group in the 2 position (Figure 1), creating more steric hindrance around the phenol, this would suggest that steric hindrance around the phenol has more of an affect with assays involving biofilms. Steric hindrance may play two different roles here; inhibiting permeation through the biofilm extracellular matrix, and obstruction of the alcohol group. These also may contribute to the observed decrease in potency with 2-allyl and 4-allyl derivatives against biofilms when compared to their parent compounds. The addition of an allyl group does slightly increase polarity, which may also inhibit the compounds ability to permeate through the biofilm matrix.

In the case of eugenol (**1b**), the 2-allyl derivative, “ortho-eugenol” (**3b**) was more potent in inhibiting planktonic cells of *S. epidermidis* although they shared the same BEC against *S. epidermidis*. **3b** also had lower BEC with *P. aeruginosa* with both planktonic cells and biofilms. This observation was in accordance with the trend found in thymol (**1a**) and carvacrol (**2a**) in which allyl derivatives were less affective against biofilms.

In this study, mammalian cells were not evaluated. Although oral LD_50_ (median lethal dose) for carvacrol and thymol has been calculated in rats to be 810 mg/kg body weight and 980 mg/ kg body weight, respectively (Jenner et al., 1964). Cytotoxicity of carvacrol and thymol was also evaluated against intestinal cells (Caco-2), finding no cytotoxic effects of thymol although carvacrol was found to cause cell death (Llana-Ruiz-Cabello et al., 2014). In a study by Machado et al. (2011) concluded that eugenol did not exhibit cytotoxicity *in vitro* toward mammalian cells at the IC_50_ determined for growth inhibition for *G. lamblia*.

Various structure activity relationships of antimicrobial compounds, including natural products and plant metabolites, and their potency toward planktonic cells and biofilms have been evaluated (Huigens et al., 2007; Richards et al., 2008, 2009; Catto et al., 2015; Garrison et al., 2015; Peeters et al., 2016; Yang et al., 2016; Choi et al., 2017; Gill et al., 2017). Richards et al. (2008) synthesized and assayed a 50-compound library of oroidin-based natural products for their anti-biofilm activity against two strains of *P. aeruginosa*, classifying several compounds as inhibitors of biofilm formation.

Structural factors such as stereochemistry, alkyl chain length, and substitution patterns have also been examined in the context of biofilms (Huigens et al., 2007; Choi et al., 2017; Gill et al., 2017). No uniform correlation of biofilm potency with planktonic potency is evident. Some studies show equipotent activity of compounds against planktonic cells and biofilms (Spoering and Lewis, 2001; Garrison et al., 2015), while other reports provide support that biofilms are more resistant to antimicrobials than planktonic cells (Costerton et al., 1999; Anderl et al., 2000; Donlan, 2001; Parsek and Singh, 2003) and still others have found that compounds exhibiting an increase in activity against planktonic cells also show increased potency against biofilms (Gill et al., 2017). The present study demonstrates that essential oil derivatives exhibiting greater activity against planktonic cells were often less effective when tested against biofilms.

## Conclusion

The presence of an allyl group in either the 2 or 4 position relative to the hydroxy phenol increases the potency of the small, phenolic essential oils thymol (**1a**) and carvacrol (**2a**) when evaluated against planktonic cells of both the Gram-positive *S. epidermidis* and the Gram-negative *P. aeruginosa*. In contrast, when the same compounds were evaluated against biofilms, the parent compounds were more potent. Similarly, eugenol (**3a**) which has an allyl group in the 4 position, was more potent than guaiacol (**3c**) against *S. epidermidis* in planktonic cell inhibition assays although less effective in killing planktonic cells and biofilms. The 2-methallyl derivatives (**1d, 2d**, and **3g**) evaluated against planktonic cells were in all cases less potent than allyl; and when compared to propyl derivatives, were less potent the majority of the time. This study illustrates the importance of using biofilm assays to determine structure-activity relationships of antimicrobials when the end target is a biofilm.

## Data Availability Statement

All datasets generated for this study are included in the manuscript/Supplementary Files.

## Author Contributions

DW performed chemical synthesis and biological evaluation against biofilm and planktonic cells with support and project supervision from TL and PS. MM performed CDC biofilm reactor assays with support from DG. The manuscript was written by DW with input from all authors.

### Conflict of Interest

The authors declare that the research was conducted in the absence of any commercial or financial relationships that could be construed as a potential conflict of interest.
