# Supplementary material for: Antimicrobial Activity of Naturally Occurring Phenols and Derivatives Against Biofilm and Planktonic Bacteria
Source: Front Chem. 2019 Oct 1;7:653. doi: 10.3389/fchem.2019.00653 (PMC6779693; doi:10.3389/fchem.2019.00653)
Supplement: Supplementary file 1 [file Data_Sheet_1.pdf]

Materials and Methods

Reactions employed oven-dried glassware under nitrogen unless otherwise noted. All reagents and starting materials were purchased from commercial suppliers and used as received, unless otherwise stated. External oil bath temperatures were used to record reaction mixture temperatures. Solvents for filtrations, transfers, and chromatography were certified ACS grade. Thin layer chromatography was performed on Silicycle Glass Backed TLC plates, and visualization was accomplished with UV light (254 nm), and/or potassium permanganate. All ^{1}H NMR spectra were recorded on a Bruker DRX300. Chemical shifts (δ) are ^{13}C NMR were recorded on a Bruker DRX500 reported in ppm, employing the solvent resonance as the internal standard. Splitting patterns are designated as: s (singlet), d (doublet), app d (apparent doublet), t (triplet), q (quadruplet), qu (quintet) dd (doublet of doublets), ddd (double double doublet), dddd (double, double, double, doublet), sept (septuplet), tq (triplet of quadruplets) and m (multiplet).

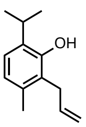

1b

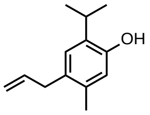

1c

Preparation of 2-(2-propen-1-yl)-6-(1-methylethyl)-3-methylphenol and 4-(2-propen-1-yl)-6-(1-methylethyl)-3-methylphenol (1b and 1c). A 25 mL round-bottom flask equipped with a magnetic stirring bar was charged with thymol (5 mmol, 1 equiv) and anhydrous acetone (5 mL) was added. Finely pulverised potassium carbonate (1.4 g, 10 mmol, 2 equiv) was then added at room temperature with stirring. The reactant mixture was heated to reflux and the allyl bromide (6 mmol, 1.2 equiv) was added. The reactant mixture was heated to reflux for 5 h. The resulting mixture was cooled and filtered through celite, washed with brine and concentrated in vacuo. The crude phenyl ether was dissolved in N,N-diethylaniline (2 mL) and heated to 200 ºC with stirring for 12 h. N,N-diethylaniline was subsequently removed by washing the mixture with 10% sulfuric acid and extracting with ethyl acetate. The residue was purified via column chromatography (25% EtOAc/Hexane for eluation) to give two different fractions, **1b** and **1c**, both as light yellow oils. ^{1}H NMR data taken in $CDCl_3$ and analytical data included the following. ^{1}H NMR (300 MHz, $CDCl_3$) **1b**: δ 6.98 (d, *J* = 7.82 Hz, 1H), 6.77 (d, *J* = 7.82 Hz, 1H), 5.95 (m, 1H), 5.12 (m, 2H), 4.93 (s, 1H) 3.44 (d, *J* = 5.88 Hz, 2H), 3.16 (sept, *J* = 6.87 Hz, 1H) 2.26 (s, 3H), 1.24 (d, *J* = 6.87 Hz, 6H). C^{13} NMR (500 MHz, $CDCl_3$) **1b**: δ 19.2 (CH_2), 22.9 (CH_3), 26.8 (CH_2), 29.3 (CH), 115.6 (CH_2), 124.3 (C), 128.8 (CH), 132.1 (C), 134.7 (CH), 137.3 (CH), 139.4 (C), 150.7 (C). H^{1} NMR (300 MHz, $CDCl_3$). ^{1}H NMR (300 MHz, $CDCl_3$) **1c**: δ 6.99 (s, 1H), 6.75 (s, 1H), 5.73 (m, 1H), 4.8 (m, 2H), 3.13 (app d, *J* = 6.1 Hz, 2H) 2.96 (sept, *J* = 6.97 Hz, 1H), 2.29 (s, 3H) 1.21 (d, *J* = 6.97 Hz, 6H). C^{13} NMR (500 MHz, $CDCl_3$) **1c**: δ 19.1 (CH_3), 23.12 (CH_3), 26.83 (CH), 33.81 (CH_2), 112.94 (CH_2), 123.53 (C), 128.91 (CH), 131.09 (CH), 134.76 (C), 137.5 (C), 143.48 (C), 152.31 (C).

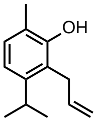

2b

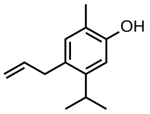

2c

Preparation of 2-(2-propen-1-yl)-3-(1-methylethyl)-6-methylphenol and 4-(2-propen-1-yl)-3-(1-methylethyl)-6-methylphenol (2b and 2c). A 25 mL round-bottom flask equipped with a magnetic stirring bar was charged with carvacrol (5 mmol, 1 equiv) and

anhydrous acetone (5 mL) was added. Finely pulverised potassium carbonate (1.4 g, 10 mmol, 2 equiv) was then added at room temperature with stirring. The reactant mixture was heated to reflux and the allyl bromide (6 mmol, 1.2 equiv) was added. The reactant mixture was heated to reflux for 5 h. The resulting mixture was cooled and filtered through celite, washed with brine and concentrated in vacuo. The crude phenyl ether was dissolved in N,N-diethylaniline (2 mL) and heated to 200 ºC with stirring for 12 h. N,N-diethylaniline was subsequently removed by washing the mixture with 10% sulfuric acid and extracting with ethyl acetate. The residue was purified via column chromatography (25% EtOAc/Hexane for eluation) to give two different fractions, **2b** and **2c**, both as yellow oils. ^{1}H NMR data taken in $CDCl_3$ and analytical data included the following. ^{1}H NMR (300 MHz, $CDCl_3$) **2b**: δ 7.01 (d, *J* = 7.88 Hz, 1H), 6.81 (d, *J* = 7.88 Hz, 1H), 5.94 (m, 1H), 5.10 (m, 2H), 4.82 (s, 1H) 3.46 (app d, *J* = 5.67 Hz, 2H), 3.07 (sept, *J* = 6.84 Hz, 1H) 2.20 (s, 3H), 1.19 (d, *J* = 6.84 Hz, 6H). C^{13} NMR (500 MHz, $CDCl_3$) **2b**: δ 15.6 (CH_3), 23.56 (CH_3), 29.59 (CH_2), 29.73 (CH), 115.47 (CH_2), 121.3 (C), 122.34 (CH), 125.28 (C), 136.76 (CH), 137.3 (CH), 139.25 (C), 151.63 (C). H^{1} NMR (300 MHz, $CDCl_3$) **2c**: δ 6.85 (s, 1H), 6.67 (s, 1H), 5.93 (m, 1H), 4.97 (m, 2H), 4.75 (s, 1H), 3.31 (app d, *J* = 6.12 Hz, 2H), 3.05 (sept, *J* = 6.87 Hz, 1H) 2.17 (s, 3H), 1.16 (d, *J* = 6.87 Hz, 6H). C^{13} NMR (500 MHz, $CDCl_3$) **2c**: δ 15.8 (CH_3), 22.42 (CH_3), 29.83 (CH), 33.76 (CH_2), 112.94 (CH2), 121.53 (C), 122.4 (CH), 125.60 (CH), 136.06 (C), 139.54 (C), 143.46 (C), 152.31(C).

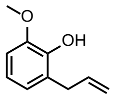

3b **Preparation of 2-(2-propen-1-yl)-6-methoxyphenol (3b).** A 25 mL round-bottom flask equipped with a magnetic stirring bar was charged with guaiacol (5 mmol, 1 equiv) and anhydrous acetone (5 mL) was added. Finely pulverised potassium carbonate (1.4 g, 10 mmol, 2 equiv) was then added at room temperature with stirring. The reactant mixture was heated to reflux and allyl bromide (6 mmol, 1.2 equiv) was added. The reactant mixture was heated to reflux for 5 h. The resulting mixture was cooled and filtered through celite, washed with brine and concentrated in vacuo. The crude phenyl ether was dissolved in N,N-diethylaniline (2 mL) and heated to 200 ºC with stirring for 12 h. N,N-diethylaniline was subsequently removed by washing the mixture with 10% sulfuric acid and extracting with ethyl acetate. The residue was purified via column chromatography (25% EtOAc/Hexane for eluation) to give **3b** as a light yellow oil. ^{1}H NMR data taken in $CDCl_3$ and analytical data included the following. H^{1} NMR (300 MHz, $CDCl_3$) **3b**: δ 6.95 (t, *J* = 8.61 Hz, 8.07, 1H), 6.79 (d, *J* = 8.07 Hz, 2.76, 1H), 6.64 (d, *J* = 8.61 Hz, 2.76, 1H), 5.66 (m, 1H), 4.86 (m, 2H) 3.69 (s, 3H), 3.24 (d, *J* = 6.29 Hz, 2H). C^{13} NMR (500 MHz, $CDCl_3$) **3b**: δ 33.9 (CH_2), 56.16 (CH_3), 109.02 (CH), 115.39 (CH_2), 122.08 (CH), 125.55 (CH), 131.13 (C), 136.73 (CH), 141.61 (C), 146.29 (C).

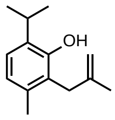

1d **Preparation of 2-methallyl-6-isopropyl-3-methyl phenol (1d).** A 25 mL round-bottom flask equipped with a magnetic stirring bar was charged with thymol (5 mmol, 1 equiv) and anhydrous acetone (5 mL) was added. Finely pulverised potassium carbonate (1.4 g, 10 mmol, 2 equiv) was then added at room temperature with stirring. The reactant mixture was heated to reflux and methallyl chloride (6 mmol, 1.2 equiv) was added. The reactant mixture was

heated to reflux for 5 h. The resulting mixture was cooled and filtered through celite, washed with brine and concentrated in vacuo. The crude phenyl ether was dissolved in N,N-diethylaniline (2 mL) and heated to 200 ºC with stirring for 12 h. N,N-diethylaniline was subsequently removed by washing the mixture with 10% sulfuric acid and extracting with ethyl acetate. The residue was purified via column chromatography (25% EtOAc/Hexane for eluation) to give **1d** as a light yellow oil. ^{1}H NMR data taken in $CDCl_3$ and analytical data included the following. H^1 NMR (300 MHz, $CDCl_3$) **1d**: δ 6.99 (d, *J* = 7.82 Hz, 1H), 6.75 (d, *J* = 7.82 Hz, 1H), 4.86 (s, 1H), 4.70 (s, 1H), 3.37 (s, 2H), 2.93 (sept, *J* = 6.87 Hz, 1H) 3.26 (s, 3H), 1.59 (s, 3H) 1.23 (d, *J* = 6.87 Hz, 6H). C^{13} NMR (500 MHz, $CDCl_3$) **1d**: δ 19.1 (CH_3), 22.42 (CH_3), 23.12 (CH_3), 26.83 (CH), 33.81 (CH_2), 112.94 (CH_2), 123.53 (C), 128.91 (CH), 131.09 (CH), 134.76 (C), 137.4 (C), 143.5 (C), 157.4 (C).

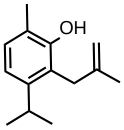

2d **Preparation of 2-methallyl-3-isopropyl-6-methyl phenol (2d).** A 25 mL round-bottom flask equipped with a magnetic stirring bar was charged with carvacrol (5 mmol, 1 equiv) and anhydrous acetone (5 mL) was added. Finely pulverised potassium carbonate (1.4 g, 10 mmol, 2 equiv) was then added at room temperature with stirring. The reactant mixture was heated to reflux and methally chloride (6 mmol, 1.2 equiv) was added. The reactant mixture was heated to reflux for 5 h. The resulting mixture was cooled and filtered through celite, washed with brine and concentrated in vacuo. The crude phenyl ether was dissolved in N,N-diethylaniline (2 mL) and heated to 200 ºC with stirring for 12 h. N,N-diethylaniline was subsequently removed by washing the mixture with 10% sulfuric acid and extracting with ethyl acetate. The residue was purified via column chromatography (25% EtOAc/Hexane for eluation) to give **1d** as a light yellow oil. ^{1}H NMR data taken in $CDCl_3$ and analytical data included the following. H^1 NMR (300 MHz, $CDCl_3$) **2d**: δ 6.99 (d, *J* = 7.86 Hz, 1H), 6.81 (d, *J* = 7.86 Hz, 1H), 4.84 (s, 1H), 4.60 (s, 1H), 3.39 (s, 2H), 3.06 (sept, *J* = 6.81 Hz, 1H) 2.19 (s, 3H), 1.80 (s, 3H), 1.17 (d, *J* = 6.81 Hz, 6H). C^{13} NMR (500 MHz, $CDCl_3$) **2d**: δ 15.1 (CH_3), 22.42 (CH_3), 23.59 (CH_3), 29.83 (CH), 33.81 (CH_2), 112.94 (CH2), 121.53 (C), 122.91 (CH), 126.60 (CH), 136.06 (C), 139.54 (C), 143.46 (C), 150.61 (C).

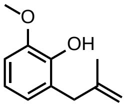

3g **Preparation of 2-methallyl-6-methoxy phenol (3g).** A 25 mL round-bottom flask equipped with a magnetic stirring bar was charged with guaiacol (5 mmol, 1 equiv) and anhydrous acetone (5 mL) was added. Finely pulverised potassium carbonate (1.4 g, 10 mmol, 2 equiv) was then added at room temperature with stirring. The reactant mixture was heated to reflux and methallyl chloride (6 mmol, 1.2 equiv) was added. The reactant mixture was heated to reflux for 5 h. The resulting mixture was cooled and filtered through celite, washed with brine and concentrated in vacuo. The crude phenyl ether was dissolved in N,N-diethylaniline (2 mL) and heated to 200 ºC with stirring for 12 h. N,N-diethylaniline was subsequently removed by washing the mixture with 10% sulfuric acid and extracting with ethyl acetate. The residue was purified via column chromatography (25% EtOAc/Hexane for eluation) to give **3g** as a light

yellow oil. ^{1}H NMR data taken in $CDCl_3$ and analytical data included the following. H^{1} NMR (300 MHz, $CDCl_3$) 3g: δ 6.95 (d, J = 7.88 Hz, 1H), 6.79 (m, 3H), 5.71 (s, 1H), 4.83 (s, 1H), 4.72 (s, 1H), 3.90 (s, 3H) 3.39 (s, 2H), 1.77 (d, 3H). C^{13} NMR (500 MHz, $CDCl_3$) 3g: δ 22.40 (CH_3), 37.58 (CH_2), 55.99 (CH_3), 108.60 (CH_3), 111.28 (CH_2), 119.24 (CH), 122.81 (CH), 125.52 (C), 143.76 (C), 144.71 (C), 146.42 (C).

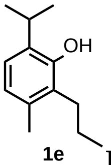

1e **Preparation of 2-*n*-propyl-6-isopropyl-3-methyl phenol (1e).** A 10 mL round-bottom flask was charged with 10% Pd/C (30 mg, 0.28 mmol, 0.1 equiv). The round-bottom flask was put under an atmosphere of hydrogen and 100% ethanol (2 mL) was added. 1b (12 mmol, 1 equiv) was added at room temperature and the reaction was allowed to stir for 12 hrs. The resulting mixture was filtered through silica and concentrated in vacuo. ^{1}H NMR data taken in $CDCl_3$ and analytical data included the following. ^{1}H NMR (300 MHz, $CDCl_3$) **1e**: δ 6.95 (d, J = 7.88 Hz, 1H), 6.81 (d, J = 7.88 Hz, 1H), 2.98 (sept, J = 6.81 Hz, 1H), 2.56 (t, J = 7.89 Hz, 2H), 2.25 (s, 3H) 1.59 (q, J = 7.89, 7.32 Hz, 2H), 1.36 (d, J = 6.81 Hz, 6H) 0.96 (t, J = 7.32 Hz, 3H). C^{13} NMR (500 MHz, $CDCl_3$) **1e**: δ 14.5 (CH_3), 19.4 (CH_3), 22.3 (CH_3), 22.7 (CH_2), 27.1 (CH_2), 28.8 (CH_2), 122.3 (CH), 123.1 (C), 126.5 (CH), 131.3 (CH), 134.7 (C), 150.8 (C).

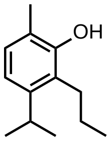

2e **Preparation of 2-*n*-propyl-3-isopropyl-6-methyl phenol (2e).** A 10 mL round-bottom flask was charged with 10% Pd/C (30 mg, 0.28 mmol, 0.1 equiv). The round-bottom flask was put under an atmosphere of hydrogen and 100% ethanol (2 mL) was added. 2b (12 mmol, 1 equiv) was added at room temperature and the reaction was allowed to stir for 12 hrs. The resulting mixture was filtered through silica and concentrated in vacuo. ^{1}H NMR data taken in $CDCl_3$ and analytical data included the following. ^{1}H NMR data taken in $CDCl_3$ and analytical data included the following. ^{1}H NMR (300 MHz, $CDCl_3$) **2e**: δ 6.95 (d, J = 7.86 Hz, 1H), 6.78 (d, J = 7.86 Hz, 1H), 3.09 (sept, J = 6.81 Hz, 1H), 2.61 (t, J = 7.95 Hz, 2H), 2.19 (s, 1H) 1.53 (q, J = 7.95, 7.29 Hz, 2H), 1.15 (d, J = 6.81 Hz, 6H), 1.0 (t, J = 7.29 Hz, 6H). C^{13} NMR (500 MHz, $CDCl_3$) **2e**: δ 14.50 (CH_3), 15.81 (CH_3), 23.55 (CH_3), 24.22 (CH_2), 27.81 (CH_2), 28.83 (CH_2), 117.17 (CH), 119.81 (C), 125.49 (CH), 128.12 (CH), 146.13 (C), 151.64 (C).

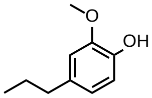

3d **Preparation of 2-*n*-propyl-2-methoxy (3d).** A 10 mL round-bottom flask was charged with 10% Pd/C (30 mg, 0.28 mmol, 0.1 equiv). The round-bottom flask was put under an atmosphere of hydrogen and 100% ethanol (2 mL) was added. Eugenol (3a) (12 mmol, 1 equiv) was added at room temperature and the reaction was allowed to stir for 12 hrs. The resulting mixture was filtered through silica and concentrated in vacuo. ^{1}H NMR data taken in $CDCl_3$ and analytical data included the following. ^{1}H NMR data taken in $CDCl_3$ and analytical data included the following. H^{1} NMR (300 MHz, $CDCl_3$) **3d**: δ 6.79 (d, J = 8.58 Hz, 1H), 6.66

(m, 2H), 3.86 (s, 3H), 2.49 (t, *J* = 7.63, 2H), 1.60 (m, J = 7.63, 7.32, 2H), 1.22 (t, J = 7.32, 3H). C^{13} NMR (500 MHz, $CDCl_3$) **3d**: δ 12.49 (CH_3), 24.67 (CH_2), 37.47 (CH_2), 56.16 (CH_3), 110.86 (CH), 114.89 (CH), 123.59 (CH), 135.39 (C), 147.13 (C), 147.81 (C).

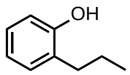

3f

Preparation of 2-*n*-propyl phenol (3f). A 10 mL round-bottom flask was charged with 10% Pd/C (30 mg, 0.28 mmol, 0.1 equiv). The round-bottom flask was put under an atmosphere of hydrogen and 100% ethanol (2 mL) was added. 3e (12 mmol, 1 equiv) was added at room temperature and the reaction was allowed to stir for 12 hrs. The resulting mixture was filtered through silica and concentrated in vacuo. ^{1}H NMR data taken in $CDCl_3$ and analytical data included the following. ^{1}H NMR data taken in $CDCl_3$ and analytical data included the following. H^1 NMR (300 MHz, $CDCl_3$) **3f**: δ 7.08 (m, 2H), 6.88 (t, *J* = 7.41 Hz, 1H), 6.74 (d, *J* = 7.89 Hz, 1H), 4.58 (s, 1H), 3.56 (t, *J* = 7.7 Hz, 2H) 1.64 (sep, *J* = 7.7, 7.34 Hz, 2H), 0.98 (t, *J* = 7.34 Hz, 3H). C^{13} NMR (500 MHz, $CDCl_3$) **3f**: δ 12.39 (CH_3), 23.18 (CH_2), 31.97 (CH_2), 116.11 (CH), 120.7 (CH), 128.69 (CH), 130.32 (C), 131.89 (C), 153.39 (C).
